# Control of Liquid-Absorbing Structure to Improve Performance of Transpiration-Type Thermoelectric Power-Generating Device Using Carbon Nanotube Composite Paper

**DOI:** 10.3390/nano15241893

**Published:** 2025-12-17

**Authors:** Kazuhide Yakata, Yuma Morita, Koya Arai, Takahide Oya

**Affiliations:** 1 College of Engineering Science, Yokohama National University, 79-5, Tokiwadai, Hodogaya-Ku, Yokohama 240-8501, Japan; 2 Mitsubishi Materials Corporation, 3-2-3, Marunouchi, Chiyoda-Ku, Tokyo 100-8117, Japan; 3 SQIE, Institute for Multidisciplinary Sciences, Yokohama National University, 79-5, Tokiwadai, Hodogaya-Ku, Yokohama 240-8501, Japan

**Keywords:** carbon nanotube (CNT), thermoelectric power generation, CNT composite paper (CNTCP), transpiration, capillary action, heat of vaporization, inner structure control

## Abstract

We propose an improved transpiration-type thermoelectric power-generating paper (t-TEPGP) with controlled water absorption. We have succeeded in developing t-TEPGP based on the carbon nanotube (CNT) composite paper (CNTCP). The CNTCP that was developed in our previous study is a composite material made from CNTs and pulp, enabling thermoelectric power generation due to its CNT content. Furthermore, CNTCP can spontaneously generate a temperature difference without requiring an external heat source, as it utilizes both the liquid absorption capacity via capillary action and the latent heat of vaporization released when the liquid evaporates. This study aimed to control the generated temperature gradient by partially modifying the internal structure of CNTCP during its manufacturing process—specifically, by altering its water absorption capacity through the choice of hot pressing or oven drying. Pressing was expected to reduce water absorption, while oven drying was predicted to increase it. Through multiple experiments, we confirmed that a sample spontaneously generated a maximum temperature difference of 0.6 °C due to evaporation heat, producing an electromotive force (E.M.F.) of 47 μV. This performance was approximately twice that of previous studies. Furthermore, we confirmed that a sample consisting of three pairs could generate an E.M.F. of 193 μV with a temperature difference of 1.2 °C.

## 1. Introduction

In recent years, as the quality of our lives has improved, much energy has been consumed. However, the energy consumed is largely wasted as heat is released, and combined with the depletion of energy resources, this has become a serious environmental problem [1,2]. As a solution to this problem, renewable energies such as solar, wind, geothermal, and vibration energies are gaining attention [3,4,5]. These renewable energies are continuously replenished in nature, allowing them to be utilized without concern for depletion. Furthermore, thermoelectric power generation technology, which converts the above-mentioned waste heat into electricity, is being studied [2,6,7]. Currently, bismuth and tellurium are used as representative materials for thermoelectric power generation; however, these are rare metals that are toxic, fragile, and heavy [8,9,10,11,12,13]. To solve the above problems, we developed a thermoelectric power generation material based on carbon nanotube (CNT) composite paper (CNTCP) [14], which is composed of previously developed CNTs and pulp (raw material of paper), at the first step of our study [15,16].

CNTs are known to show high electrical and thermal conductivities [17,18] as well as high thermo-electromotive force (t-E.M.F.) [19]. E.M.F. observed from thermoelectric materials for each temperature difference of 1 K (1 °C) is called the Seebeck coefficient. The Seebeck coefficient of bismuth and tellurium alloys has been shown to be 200 μV/K, while that of CNTs is 170 μV/K [19]. CNTs are generally in powder form and difficult to handle, so they are primarily used as composite materials by combining them with other materials. Many practical CNT-based materials have been developed, including buckypapers [20], such as CNT-based thermoelectric materials [21]. Many unique and useful devices have also been developed using CNTCPs, including triboelectric nanogenerators [22], soft actuators [23], and electromagnetic shielding [24,25].

As the second step of our study, we succeeded in developing the “transpiration-type thermoelectric power-generating paper” [26] that works by combining the “capillary action” [27,28] of paper [29,30,31], the base material of CNTCP, with the “heat of vaporization” (HoV) [32] of water absorbed by the capillary action. In nature, plants absorb water from the ground during transpiration [33,34,35,36,37] and use HoV when releasing water from their leaves [38,39,40]. In other words, plants create temperature differences through transpiration. Similarly, in this study, CNTCP can spontaneously produce a temperature difference, thus realizing thermoelectric power generation without the need for a heat source. This is the world’s first thermoelectric power generation method realized by utilizing CNTCP, which has the functions of both CNT and paper.

In our previous study [26], we found that the water absorption ability of CNTCP can be easily controlled by changing the drying and molding process within the fabrication method. In other words, depending on the drying and molding process, it is possible to produce samples that can generate HoV and samples that do not generate HoV. In this study, we found that different drying and forming methods can be applied simultaneously to the same sample. This makes it possible to create a part with water absorption ability and a part without water absorption ability in the same sample, and it was found that it is possible to improve t-E.M.F. from the same sample. In the following sections, we report on the newly developed fabrication method and the improvement in t-E.M.F. of CNTCP.

## 2. Materials and Methods

### 2.1. CNTCP Fabrication Method

**Figure 1:**
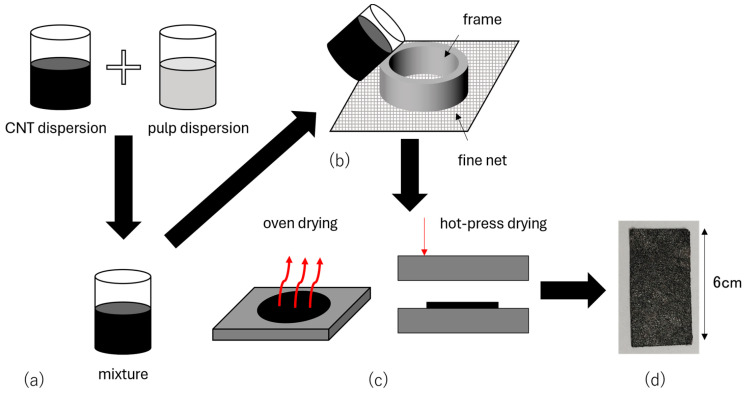
CNTCP fabrication (paper-making) method. (**a**) Preparing CNT-pulp mixed dispersion; (**b**) straining water with fine net; (**c**) drying methods: oven drying or hot-press drying; and (**d**) completing CNTCP samples.

The fabrication of CNTCP follows the traditional Japanese *washi* paper-making process, as shown in Figure 1 [14,16]. Figure 1a illustrates the process leading to the preparation of the mixture obtained by mixing the CNT dispersion and the pulp dispersion. To prepare the CNT dispersion, 22 mg of single-walled CNTs (ZEONANO SG101, Zeon Corporation, Tokyo, Japan, diameter = 2–3 nm, length = 300–500 μm) and 150 mg of sodium dodecyl sulfate (SDS) as the dispersant are added to 20 mL of pure water, and the mixture is ultrasonicated for 1 h. It is generally known that there is a maximum concentration at which CNTs dispersed in water can exist stably. In contrast, in our fabrication process, we use a large amount of CNTs because our approach differs from the conventional approach to CNT dispersion preparation. Generally, it is considered desirable for CNTs in a CNT dispersion to be isolated. However, our approach does not require complete isolation of CNTs in water. While isolated CNTs may offer greater potential for utilizing their performance effectively, it is found that a certain degree of bundling is preferable for CNT fixation to paper fibers. Even if CNTs exist in such large quantities within the dispersion that they cannot be sufficiently dispersed theoretically, the coexistence of paper fibers in the dispersion allows CNTs to adhere to the paper fibers in quantities exceeding the theoretical amount, enabling their utilization by the paper fibers. Another reason is that during the paper-making process, not all prepared CNTs are fixed to the paper; some inevitably leach out during dewatering. The quantity prepared accounts for this yield issue. Other studies have reported adding flocculants to the mixed dispersion of paper fibers and CNTs to address this yield issue. The determination of CNT usage is based on results from our past research; however, it is expected that reducing usage will be possible once yield issues are resolved in the near future. In our previous study, we observed that SG101 exhibited a good Seebeck coefficient of approximately 30 μV/K in CNTCP, and, therefore, we use it in this study. For the pulp dispersion, 150 mg of rayon is dispersed in 100 mL of pure water and stirred for 1 h. Rayon is found to be highly absorbent and appropriate for CNTCP as the transpiration-type thermoelectric power-generating paper. Next, as shown in Figure 1b, we mix these dispersions and dehydrate using a fine net. Finally, we dry the CNTCP to form it into paper, as illustrated in Figure 1c. There are two drying methods for the CNTCP preparation: hot-press drying (HPD) and oven drying (OD). The temperature setting for HPD was set to 100 °C, and, for OD, it was set to 80 °C. We chose these settings because of the devices used and the heat-resistant temperature of the paper. Regarding the pressure of the HPD, precise values cannot be determined due to equipment limitations; however, according to the specifications, it can apply a maximum load of approximately 1 MPa. We believe that a load close to this value was applied in this study as well. As described later, we select these two drying processes according to the purpose and prepare the desired samples. The CNTCP fabricated with the above quantities is cut into a 6.0 cm × 3.0 cm sheet, as shown in Figure 1d.

### 2.2. Evaluation of Thermoelectric Power-Generating Performance of Samples with Different Drying Methods

**Figure 2:**
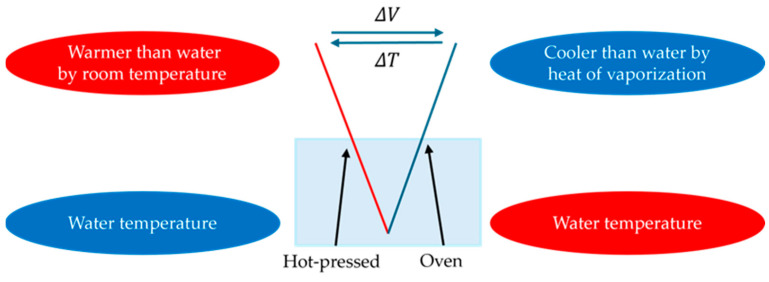
Schematic of CNTCP samples subjected to different drying methods and immersed in water.

To evaluate the influence of different drying methods on transpiration-type thermoelectric power generation, we prepare three types of CNTCPs: a hot-press-dried CNTCP (HPD-CNTCP), an oven-dried CNTCP (OD-CNTCP), and a halved CNTCP consisting of half hot-press-dried and half oven-dried areas on a single CNTCP (HPDOD-CNTCP) (Table 1). Then, we prepare 6.0 cm × 3.0 cm samples in the above-mentioned fabrication method. HPD-CNTCP is a sample where the whole sample is dried by HPD, and OD-CNTCP is a sample where the whole sample is dried by OD-CNTCP. HPDOD-CNTCP is a sample where half of the sample (3.0 cm × 3.0 cm) is subjected to HPD, while the other half is OD. As a comparative experiment, the central part of each of the three types of CNTCPs described above is folded and immersed in pure water, while both ends are fixed in air, as shown in Figure 2. In the case of HPOD-CNTCP, the central part serves as the boundary between the two drying methods. We measure the temperature at the left and right ends of the CNTCP and the overall generated t-E.M.F. The temperature at the left end of the sample is defined as *T*_l_ [°C] and that at the right end as *T*_r_ [°C]. The temperature difference between the left and right ends of the sample is calculated as Δ*T*, and t-E.M.F. across the sample is denoted as Δ*V*, as defined in Figure 2.

### 2.3. Experimental Method to Improve E.M.F. By Using Multisheet

**Figure 3:**
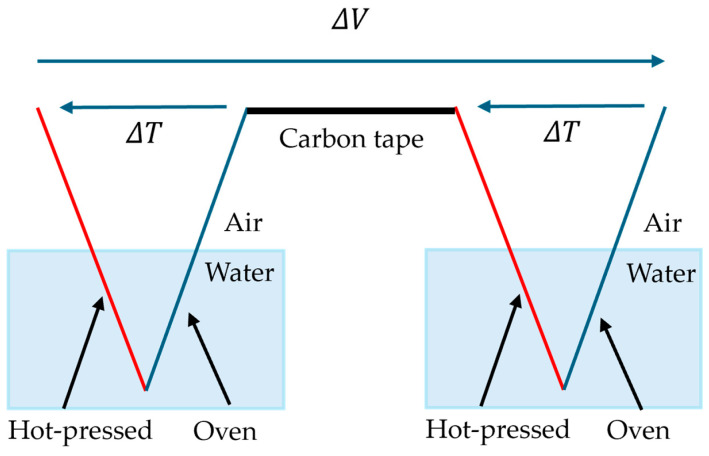
Schematic of two-sheet structures.

**Figure 4:**
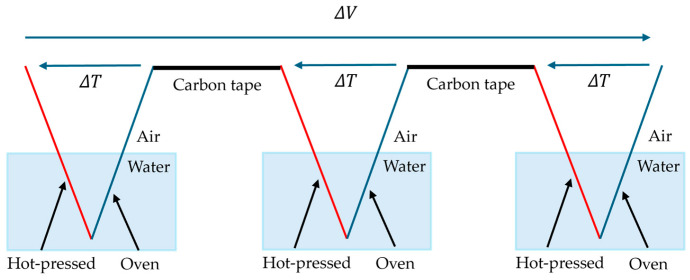
Schematic of three-sheet structures.

Generally, it is known that in thermoelectric power-generating materials, connecting multiple elements electrically in series and thermally in parallel allows greater output to be obtained from one heat source. In this study, to achieve higher output, multiple HPDOD-CNTCP samples are prepared and connected. Figure 3 shows a schematic of two-sheet structures, and Figure 4 shows a schematic of three-sheet structures. In HPDOD-CNTCP, here, the end of the HPD part of one sample is connected to the end of the OD part of another sample using carbon tape for simplicity. By connecting multiple samples in series in this way, the generation of a larger t-E.M.F. is expected.

## 3. Results and Discussions

### 3.1. Evaluation of Thermoelectric Power Generation Performance of Sample Incorporating Proposed Structure

**Figure 5:**
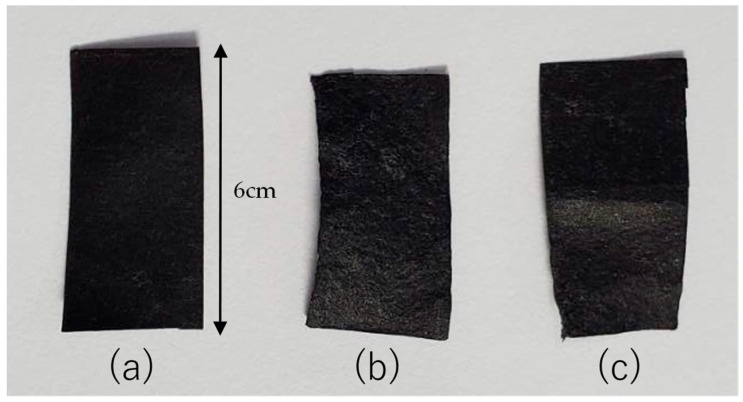
Fabricated CNTCP samples. Samples were fabricated under conditions listed in Table 1: (**a**) sample No. 1 (HPD-CNTCP), (**b**) sample No. 2 (OD-CNTCP), (**c**) sample No. 3 (HPDOD-CNTCP). Each sample was prepared as 6.0 cm × 3.0 cm sheet.

To evaluate the influence of the drying methods, we first prepared samples 1 to 3 as shown in Figure 5 based on the CNTCP fabrication method described in Section 2.1. As explained above, the samples were prepared to be 6.0 cm × 3.0 cm in size. However, Figure 5 clearly shows samples of different sizes. This difference is due to the chosen drying methods. When using HPD, the size of the paper sample change is relatively less likely to occur. On the other hand, OD causes shrinkage of the pulp (cellulose) fibers that make up the composite paper during drying. Consequently, the sample in Figure 5b appears smaller in size. However, since it is not pressed, the sample becomes thicker. Figure 5c shows the upper half pressed and the lower half unpressed, so it exhibits characteristics of both the samples in Figure 5a,b.

**Figure 6:**
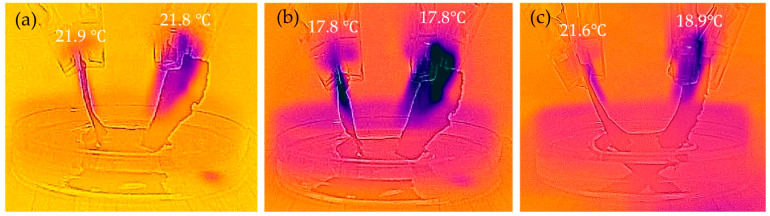
Temperature difference generation by HoV (thermal image) of samples (**a**) HPD-CNTCP, (**b**) OD-CNTCP, (**c**) HPDOD-CNTCP (water temperature: 20.5 °C; room temperature: 22.2 °C).

**Figure 7:**
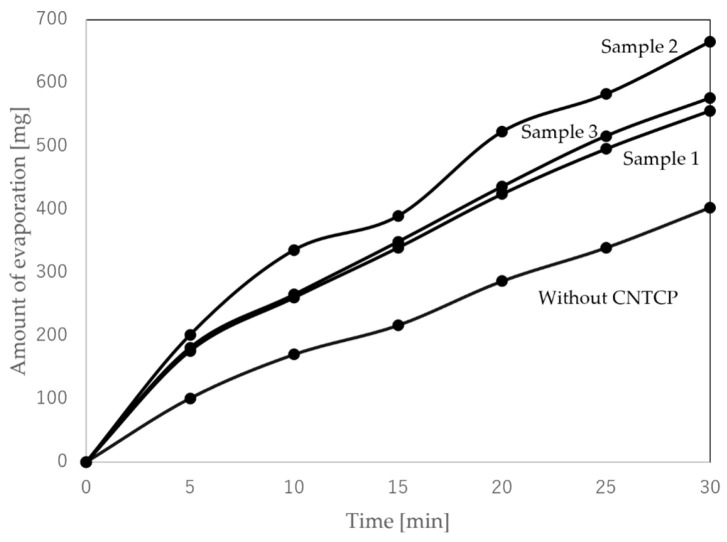
Water absorption rate of HPD-CNTCP and OD-CNTCP.

**Figure 8:**
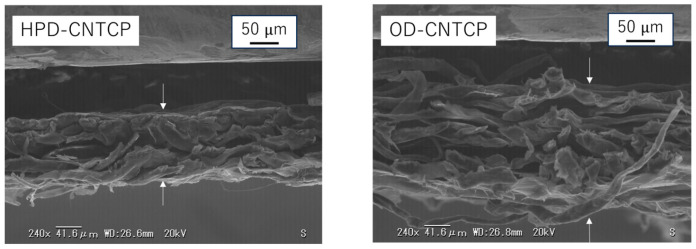
SEM images of HPD-CNTCP and OD-CNTCP (cross-sectional view). Substance between arrows is CNTCP sample, and its cross-section is being observed.

Figure 6 and Table 2 show the results of the generated temperature difference in the sample due to capillary action and the resulting HoV. The room temperature and water temperature at the start of this experiment were 22.2 °C and 20.5 °C, respectively. Additionally, the humidity during the experiment was around 60%RH, and there was no wind. For No. 1, since the drying method was HPD, capillary action occurred minimally. Consequently, no latent HoV was obtained, and both *T*_r_ and *T*_l_ were almost equivalent to room temperature. In contrast, for No. 2, since the drying method was OD, capillary action originating from internal voids occurred. Consequently, HoV was generated, and it is considered that both *T*_r_ and *T*_l_ became lower than room temperature. Both samples showed clear results due to the drying method. Figure 7 and Figure 8 show the water absorption rate and cross-sectional SEM images of the structures of the fabricated HPD-CNTCP and OD-CNTCP, respectively. Clear differences in the absorption rate, thickness, and cross-sectional structure were observed depending on the drying method. As intended, the HPD treatment compressed internal voids and reduced thickness. Consequently, the SEM images also supported the conclusion that water absorption and HoV decreased due to suppressed capillary action. In contrast, OD treatment, as expected, preserved the internal void structure and thickness, promoting capillary action. This resulted in increased water absorption and the resultant HoV, which was also supported by structural evaluation. In addition, here, noteworthy is the difference between *T*_r_ and *T*_l_. This indicates that there is almost no temperature difference between the left and right sides. Samples No. 1 and No. 2 were dried using a single method across the entire sample, resulting in symmetrical water absorption behavior centered around the fold. Therefore, when the same drying method is applied to the entire sample, a temperature difference cannot be obtained. In other words, even if there is a temperature difference between the sample end in water and the other sample end in air, the temperature gradient is identical. Therefore, even if thermoelectric conversion occurs in each part, it cancels each other out. As a result, sufficient output cannot be obtained. This is what was observed in the actual measurements. In contrast, sample No. 3 shows the water absorption behavior based on each drying method on the left and right sides, respectively. The key point to note is that, unlike the other two samples, it successfully created a 0.6 °C temperature difference. Based on these results, as proposed, applying HPD and OD to the sample in each desired area, we can obtain a single sample with areas of differing water absorption. This sample is suitable for the transpiration-type thermoelectric power generation. For more detailed characterization, it is necessary to analyze the temperature distribution along the current path. However, at present, it is extremely difficult to determine the overall configuration of the CNT network within the composite paper. This is because the CNT composite paper consists of a three-dimensional conductive network formed by integrating CNTs into the three-dimensional structure of pulp fibers. As mentioned earlier, while CNTs themselves are nanometer-diameter and several hundred micrometers long materials, within the composite paper, the presence of paper fibers causes the CNT network to form a millimeter-scale three-dimensional structure. Based on findings from our previous study [26], we assume that both the electric current and water flow are averaged across the entire sample and flow uniformly. To evaluate the temperature distribution in greater detail, it would be desirable to obtain temperature profiles by fixing multiple thermocouples to the composite paper. However, this is currently difficult. Because, for example, fixing the thermocouples to the sample using adhesive would inhibit capillary action. Similarly, problems are expected to arise even when fixing thermocouples to the sample using clips or similar methods. Because the pressure from the clip crushes the internal structure of the sample, which affects the capillary action within the sample. At present, using a higher-resolution thermography camera is considered the most practical approach. This will be clarified in future research. Furthermore, evaluation of the temperature difference using thermal images confirmed that it increases with distance from the water surface. This indicates that a certain sample length is advantageous for obtaining the temperature difference. However, it was also confirmed that simply increasing the distance from the water surface is not effective. Specifically, extending the sample length beyond a certain point does not further improve the efficiency of HoV acquisition. Additionally, the electrical resistance of the sample itself increases as the sample length increases. Future research will focus on determining the optimal sample size to efficiently obtain HoV and achieve high thermoelectric conversion performance.

**Figure 9:**
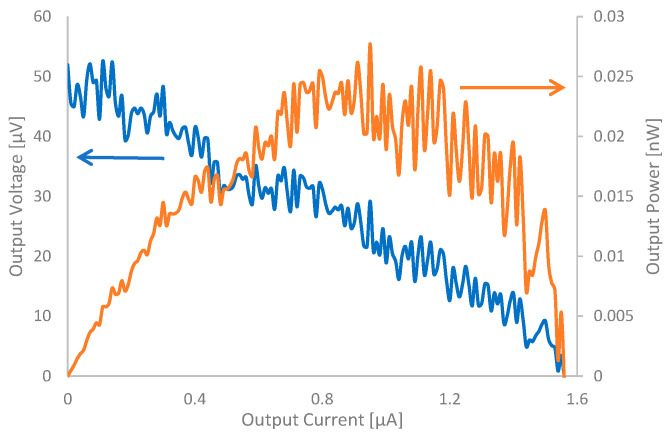
Power generation characteristics of samples. In this experiment, room temperature was 22.6 °C, water temperature was 20.7 °C, ambient humidity was 20%RH, and temperature difference Δ*T* generated by sample was 1.8 °C. Internal resistance of the sample calculated from this load curve was approximately 31.4 Ω.

Table 3 shows the t-E.M.F., calculated Seebeck coefficient, and power output results obtained from the samples. Figure 9 shows the load curve of the sample measured using a semiconducting parameter analyzer (Semiconductor Characterization System, KEITHLEY, 4200A-SCS, Solon, OH, USA). In these experiments, the internal resistance before power generation was 17.3 W in the dry condition and 18.3 W in the wet condition for No. 1, 13.4 W (dry) and 28.0 W (wet) for No. 2, and 19.5 W (dry) and 24.2 W (wet) for No. 3, respectively. Differences in resistance exist depending on the drying method used for the samples. Since this study involves manual fabrication, there is some variation. However, the thickness of the samples differs significantly depending on whether a press is used. Consequently, even if the amount of CNTs used is the same, differences in density arise, leading to differences in conductivity. The results indicate that while t-E.M.F. was almost undetectable for No. 1 and No. 2, the t-E.M.F. of 47 μV was observed in No. 3, yielding a calculated Seebeck coefficient of 78.3 μV/K. Although there may be discussions about expressing this value as the Seebeck coefficient since it combines two areas separated by a fold, this paper assumes it is the Seebeck coefficient because the material responsible for the thermoelectric performance is only one type of CNT contained in CNTCP. In our previous study [26], the Seebeck coefficient of a single CNTCP sheet prepared using SG101-CNT was approximately 40 μV/K. As described above, the Seebeck coefficient of HPDOD-CNTCP was 78.3 μV/K, approximately twice the value obtained in the previous study. This is because, even using a single type of CNT, the composite paper form and the application of HPD and OD treatments enabled effective utilization of the thermal gradient. Here, since the measurement was performed with water contained within the samples, all data should be considered as device-effective values. We also found the values to be 36.6 µV/K for HPD and 42.5 µV/K for OD when no water was used, and a temperature difference was induced by applying a heat source to one side of the samples. Therefore, we believe heat transfer losses due to water have some degree of influence. Since the Seebeck coefficient of the samples has been estimated, the next point of interest is the values of the thermoelectric figure of merit ZT and conversion efficiency of the samples. To calculate conversion efficiency, it is necessary to determine ZT. Calculating ZT requires deriving the power factor (PF) of the sample and thermal conductivity. While PF was calculated as $2.12\times 10^{-6}$ W/m·K^2^ in this study, the thermal conductivity is difficult to estimate accurately due to the unique structure of the composite paper. In our previous study [16], the thermal conductivity of the composite paper was roughly estimated to be 1.5 W/m·K, and we assume the samples in this study will have similar values. This time, the influence of water (thermal conductivity 0.60 W/m·K @ 22 °C [41]) during thermoelectric conversion further increases the difficulty of deriving the thermal conductivity. Assuming the thermal conductivity of these samples, including the water effect, is 1 W/m·K, ZT at room temperature would be $6.25\times 10^{-4}$. Therefore, the conversion efficiency is calculated to be approximately $3.17\times 10^{-5}$%. The accuracy of this estimation will be clarified in future research.

### 3.2. Examination to Improve E.M.F. By Using Multisheet

As described in the previous section, since the proposed HPDOD-CNTCP was found to be highly suitable for transpiration-type thermoelectric power generation, the next step was to investigate enhancing output by connecting these thermoelectric elements. To this end, three HPDOD-CNTCP samples were prepared, and their response was tested using the method described in Section 2.3. Table 4 shows the results of the temperature difference generated from each sample. The room temperature and water temperature at the start of this experiment were 23.9 °C and 23.2 °C for one and two sheets, and 25.8 °C and 24.8 °C for three sheets.

Table 5 shows the average temperature difference per sheet and the t-E.M.F. obtained by connecting multiple samples, and the calculated values of t-E.M.F. per degree of temperature difference. The results showed that the t-E.M.F. increases in an approximately linear manner with the number of HPDOD-CNTCPs samples used. As the same as the single sheet described in Section 3.1, the results obtained by multiple sheets can also be considered to be the effective values. Beyond the influence of water, losses such as contact resistance at the sheet connecting points and increased resistance due to shape changes caused by water absorption are also possible. Therefore, future challenges include analyzing the contact resistance between samples and realizing a shape that utilizes capillary action without adversely affecting the electrical characteristics. Based on the above measurement results, we believe the usefulness of multiple samples has been demonstrated. The load curve with the connected samples is also of interest, but clear data could not be obtained in this experiment due to measurement environment issues. This will be clarified in a future study.

## 4. Conclusions

We have developed the transpiration-type thermoelectric power-generating paper based on CNTCP through our previous research [26]. This material is a novel thermoelectric material capable of generating electricity without an external high-temperature heat source by utilizing the capillary action of paper to absorb liquid and the HoV of the absorbed liquid. The aim of this study was to enhance the t-E.M.F. of the transpiration-type thermoelectric power-generating paper by controlling its water absorption. To achieve this, we investigated modifying the drying method within the CNTCP making methods. Specifically, we explored altering the void structure within the composite paper and controlling its water absorption by changing the drying method. We examined three drying methods—HPD, OD, and HPDOD—and fabricated CNTCP based on these methods. Next, the HoV based on water absorption and the Seebeck coefficient were evaluated for these three CNTCPs. From the experiments, the fabricated sample spontaneously generated a temperature difference of 0.6 °C due to the heat of evaporation and produced an electromotive force of 47 μV. The Seebeck coefficient reached 78.3 μV/K, approximately twice that of the previous transpiration-type thermoelectric power-generating paper reported in our previous research [26]. Furthermore, to achieve more efficient thermoelectric conversion, a multisheet structure was also investigated, and confirmed that t-E.M.F. generated increased. In this study, only p-type semiconducting CNTs were used. It is known that semiconducting CNTs naturally become p-type due to the influence of oxygen in air, so no specific doping was performed in this study. As a future study, we plan to obtain n-type CNTs through doping to develop higher-performance thermoelectric conversion elements. We have already conducted n-type conversion experiments in other studies, and in those cases, we confirmed that differences in drying methods had little effect.

This transpiration-type thermoelectric power-generating paper can generate electricity without requiring an external heat source as long as liquid is available, making it an eco-friendly power supply suitable for use in natural environments such as rivers and oceans. Although challenges remain, we believe the usefulness of this research concept—a device that utilizes liquid to generate its own temperature difference for thermoelectric power generation—has been demonstrated. This research is expected to further enhance its practical feasibility.

## Figures and Tables

**Table 1:** Drying method of samples.

Sample No.	Drying Method
1	HPD
2	OD
3	Half of HPD and other half of OD

**Table 2:** Temperature difference obtained from samples.

Sample	*T*_r_ [°C]	*T*_l_ [°C]	$\Delta T\left(T_{r}-T_{l}\right)$ [°C]
1 HPD-CNTCP	25.0	24.8	0.2
2 OD-CNTCP	23.9	23.8	0.1
3 HPDOD-CNTCP	24.8	24.2	0.6

**Table 3:** Measured t-E.M.F. from samples and calculated Seebeck coefficient.

Sample No.	ΔT [℃]	ΔV [μV]	Seebeck Coefficient [μV/K]	Power Output [nW]
1	0.2	3	15	$3\times 10^{-5}$
2	0.1	1	10	$1\times 10^{-5}$
3	0.6	47	78.3	0.16

**Table 4:** Temperature difference obtained from prepared samples.

Numbers of HPDOD-CNTCPs	*T*_r_ [°C]	*T*_l_ [°C]	$\Delta T\left(T_{r}-T_{l}\right)$ [°C]	ΔV [ μV]
1	23.7	23.0	0.7	56
2	23.8	23.0	0.8	63
	23.6	23.0	0.6	52
			(Ave.: 0.7)	(Ave.: 57.5)
3	25.4	24.2	1.2	92
	25.4	24.4	1.0	78
	25.5	24.1	1.4	103
			(Ave.: 1.2)	(Ave.: 91)

**Table 5:** Obtained t-E.M.F. from multiple sheets of HPDOD-CNTCTP.

Numbers of HPDOD-CNTCPs	ΔT [°C]	ΔV [ μV]	t-E.M.F. Per 1 K [ μV/K]
1	0.7	56	80
2	0.7	109	156
3	1.2	232	193

## Data Availability

The data that support the findings of this study are available from the corresponding author upon reasonable request.

## Abbreviations

The following abbreviations are used in this manuscript:

CNT	carbon nanotube
CNTCP	carbon nanotube composite paper
SDS	sodium dodecyl sulfate
t-E.M.F.	thermo-electromotive force
HP-CNTCP	hot-pressed carbon nanotube composite paper
OD-CNTCP	oven-dried carbon nanotube composite paper
HPDOD-CNTCP	half hot-press-dried and half oven-dried carbon nanotube composite paper
HoV	heat of vaporization

## References

[1] Oyedepo S.O., Fakeye B.A. (2021). Waste heat recovery technologies: Pathway to sustainable energy development. J. Therm. Eng..

[2] Geffroy C., Lilley D., Parez P.S., Prasher R. (2021). Techno-economic analysis of waste-heat conversion. Joule.

[3] Sayed E.T., Olabi A.G., Alami A.H., Radwan A., Mdallal A., Rezk A., Abdelkareem M.A. (2023). Renewable Energy and Energy Storage Systems. Energies.

[4] Hassan Q., Algburi S., Sameen A.Z., Al-Musawi T.J., Al-Jiboory A.K., Salman H.M., Ali B.M., Jaszczur M. (2024). A comprehensive review of international renewable energy growth. Energy Built Environ..

[5] Hassan Q., Viktor P., Al-Musawi T.J., Mahmood Ali B., Algburi S., Alzoubi H.M., Al-Jiboory A.K., Sameen A.Z., Salman H.M., Jaszczur M. (2024). The renewable energy role in the global energy Transformations. Renew. Energy Focus.

[6] Snyder G., Tobere E. (2008). Complex thermoelectric materials. Nat. Mater..

[7] Caylor J., Coonley K., Stuart J., Colpitts T., Venkatasubramanian R. (2005). Enhanced thermoelectric performance in PbTe-based superlattice structures from reduction of lattice thermal conductivity. Appl. Phys. Lett..

[8] Poudel B., Hao Q., Ma Y., Lan Y., Minnich A., Yu B., Yan X., Wang D., Muto A., Vashaee D. (2008). High-Thermoelectric Performance of Nanostructured Bismuth Antimony Telluride Bulk Alloys. Science.

[9] Okuda T., Nakanishi K., Miyasaka S., Tokura Y. (2001). Large thermoelectric response of metallic perovskites: Sr_1−x_La_x_TiO_3_ (0 < ∼x < ∼0.1). Phys. Rev. B.

[10] Minnich A., Dresselhaus M., Ren Z., Chen G. (2009). Bulk nanostructured thermoelectric materials: Current research and future prospects. Energy Environ. Sci..

[11] Buge A., Supino-Viterbo V., Rancurel G., Pontes C. (1981). Epileptic phenomena in bismuth toxic encephalopathy. J. Neurol. Neurosurg. Psychiatry.

[12] Taylor A. (1996). Biochemistry of tellurium. Biol. Trace Elem. Res..

[13] Sundar S., Chakravarty J. (2010). Antimony toxicity. Int. J. Environ. Res. Public Health.

[14] Oya T., Ogino T. (2008). Production of electrically conductive paper by adding carbon nanotubes. Carbon.

[15] Kawata K., Oya T. (2017). Development and evaluation of “thermoelectric power-generating paper” using carbon nanotube-composite paper. Jpn. J. Appl. Phys..

[16] Miyama A., Oya T. (2022). Improved performance of thermoelectric power generating paper based on carbon nanotube composite papers. Carbon Trends.

[17] Ebbesen T., Lezec H., Hiura H., Bennett J., Ghaemi H., Thio T. (1996). Electrical conductivity of individual carbon nanotubes. Nature.

[18] Yu M.F., Files B., Arepalli S., Ruoff R. (2000). Tensile Loading of Ropes of Single Wall Carbon Nanotubes and their Mechanical Properties. Phys. Rev. Lett..

[19] Nakai Y., Honda K., Yanagi K., Kataura H., Kato T., Yamamoto T., Maniwa Y. (2014). Giant Seebeck coefficient in semiconducting single-wall carbon nanotube film. Appl. Phys. Express.

[20] Liu J., Rinzler A., Dai H., Hafner J., Bradley R., Boul P., Lu A., Iverson T., Shelimov K., Huffman C.B. (1998). Fullerene Pipes. Science.

[21] Blackburn J., Ferguson A., Cho C., Grunlan J. (2018). Carbon-nanotube-based thermoelectric materials and devices. Adv. Mater..

[22] Okochi K., Oya T. (2024). Unique triboelectric nanogenerator using carbon nanotube composite papers. Appl. Sci..

[23] Toyomasu R., Oya T. (2024). Development and improvement of “Paper actuator” based on carbon-nanotube composite paper with its unique structures. J. Compos. Sci..

[24] Fugetsu B., Sano E., Sunada M., Sambongi Y., Shibuya T., Wang X., Hiraki T. (2008). Electrical conductivity and electromagnetic interference shielding efficiency of carbon nanotube/cellulose composite paper. Carbon.

[25] Imai M., Akiyama K., Tanaka T., Sano E. (2010). Highly strong and conductive carbon nanotube/cellulose composite paper. Compos. Sci. Technol..

[26] Kamekawa Y., Arai K., Oya T. (2023). Development of transpiration-type thermoelectric-power-generating material using carbon nanotube composite papers with capillary action and heat of vaporization. Energies.

[27] Gennes P.G., Brochard-Wyart F., Quéré D. (2004). Capillarity and Wetting Phenomena: Drops, Bubbles, Pearls, Waves.

[28] Morrow N.R. (1970). Physics and Thermodynamics of Capillary Action in Porous Media. Ind. Eng. Chem..

[29] Gillespie T. (1959). The capillary rise of a liquid in a vertical strip of filter paper. J. Colloid Sci..

[30] Mohammadi S., Busa L.S.A., Maeki M., Mohamadi R.M., Ishida A., Tani H., Tokeshi M. (2016). Novel concept of washing for microfluidic paper-based analytical devices based on capillary force of paper substrates. Anal. Bioanal. Chem..

[31] Böhm A., Carstens F., Trieb C., Schabel S., Biesalski M. (2014). Engineering microfluidic papers: Effect of fiber source and paper sheet properties on capillary-driven fluid flow. Microfluid. Nanofluid..

[32] Haggenmacher J. (1946). The heat of vaporization as a function of pressure and temperature. J. Am. Chem. Soc..

[33] Gupta B., Shah D., Mishra B., Joshi P., Gandhi V.G., Fougat R. (2015). Effect of top soil wettability on water evaporation and plant growth. J. Colloid Interface Sci..

[34] Arkley R.J. (1963). Relationships between plant growth and transpiration. Hilgardia.

[35] Graamans L., van den Dobbelsteen A., Meinen E., Stanghellini C. (2017). Plant factories; crop transpiration and energy balance. Agric. Syst..

[36] Pearcy R.W., Schulze E.D., Zimmermann R. (2000). Measurement of transpiration and leaf conductance. Plant Physiological Ecology: Field Methods and Instrumentation.

[37] Yang Z., Sinclair T.R., Zhu M., Messina C.D., Cooper M., Hammer G.L. (2012). Temperature effect on transpiration response of maize plants to vapour pressure deficit. Environ. Exp. Bot..

[38] Gates D.M. (1968). Transpiration and Leaf Temperature. Annu. Rev. Plant Physiol..

[39] Lin H., Chen Y., Zhang H., Fu P., Fan Z. (2017). Stronger cooling effects of transpiration and leaf physical traits of plants from a hot dry habitat than from a hot wet habitat. Funct. Ecol..

[40] Gupta S., Ram J., Singh H. (2018). Comparative Study of Transpiration in Cooling Effect of Tree Species in the Atmosphere. J. Geosci. Environ. Prot..

[41] Ramires M.L.V., Nieto de Castro C.A., Nagasaka Y., Nagashima A., Assael M.J., Wakeham W.A. (1995). Standard Reference Data for the Thermal Conductivity of Water. J. Phys. Chem. Ref. Data.

